# An image, an idea: expert insights on guiding principles for promoting physical activity

**DOI:** 10.3389/fspor.2025.1640346

**Published:** 2025-12-09

**Authors:** Alberto Aibar, Eduardo Generelo Lanaspa, José Antonio Julián Clemente, Ana Corral-Abos, Gemma Bermejo, Vicente J. Beltrán-Carrillo

**Affiliations:** 1 Universidad de Zaragoza, Huesca, Spain; 2 Universidad Miguel Hernandez de Elche, Elche, Spain

**Keywords:** ecological model, salutogenic model, new materialism, empowerment, physical environment, photo-elicitation

## Abstract

This study explores the guiding principles for promoting physical activity (PA) based on the perspectives of internationally recognized experts. Using qualitative methodologies and visual techniques, including photo-elicitation, present research gathered insights from 50 experts across 13 countries. Findings highlight the importance of adopting multilevel and multicomponent approaches that integrate individual, social and environmental factors. At an individual level, motivation, enjoyment and motor competence are key for sustaining active lifestyles. Social influences, particularly from relatives, peers, educators and the media, play a critical role in fostering supportive environments that encourage PA, with gender perspectives and the needs of marginalized groups being important to also consider. Additionally, the physical environment emerged as a decisive factor, with experts stressing the importance of safe, accessible and pleasant urban spaces that promote active transportation and spontaneous play. Present findings provide a comprehensive framework for designing more effective and sustainable PA promotion strategies, ensuring that interventions move beyond isolated initiatives and become embedded within broader social, educational and political contexts. This report offers valuable insights to inspire future dialogue and collective action, ultimately contributing to healthier, more active and equitable societies.

## 1 Introduction

The promotion of physical activity (PA) has become a priority public health objective, due to the high levels of physical inactivity that currently exist in contemporary populations, alongside the negative effects of this on health and wellbeing (1). Further, physical inactivity has emerged as a leading risk factor for developing non-communicable diseases, such as obesity, type 2 diabetes and cardiovascular diseases, all of which have a negative impact on quality of life and increase the burden on public health systems. However, the complex motives that drive individual choices when opting to lead active or sedentary lifestyles, and the most effective approaches for increasing population-level PA engagement, are not yet fully understood.

The opinions and recommendations of experts in PA promotion may be of particular interest when it comes to shedding light on these issues. Experts are researchers who are recognised by their peers to be of international prestige, due to their long professional careers in PA promotion, high publication output and telling participation in research projects. The engagement of experts in PA promotion helps to identify evidence-based strategies and best practices, which enables the design of more effective and context-sensitive interventions aimed at promoting active and healthy lifestyles in the population (2). Expert recommendations can form the cornerstone for better identifying the needs that must be addressed and the challenges or barriers that must be overcome to effectively promote PA. This, in turn, will help to define basic principles for guiding more effective and sustainable interventions. Subsequently, through the establishment of clear guiding principles based on scientific evidence and expert opinion, it should be possible to design more effective and sustainable interventions that address the complex determinants of PA (3).

The salutogenic approach (4) to PA promotion emphasizes the factors that support health and well-being, as opposed to merely focusing on risk reduction. This perspective shifts the narrative from disease prevention to enhancing individual capacity to lead active and meaningful lives. For this to occur, deep consideration must be given to the factors influencing health behaviours, such as PA. The socio-ecological model allows for better understanding of the factors that influence PA and, in this way, enables a comprehensive approach by considering multiple levels of influence, including individual, interpersonal, community and policy-related factors (5). This model highlights that PA promotion requires more than simply targeting individual behaviour. Instead, it requires creating supportive environments, fostering social networks and implementing policies that facilitate active lifestyles. By addressing these interconnected layers, future interventions can be more effective, sustainable and equitable. However, for this to occur, more in-depth understanding is needed of the impact of different factors and the way in which they are interrelated.

Within the theoretical framework provided by the socioecological model, different theories or approaches, such as the self-determination theory (SDT) and the new materialism approach, are widely used by researchers and experts as they seek to understand influencing factors. At an individual level, SDT provides a robust framework for understanding the personal factors that influence PA by focusing on the fulfilment of three basic psychological needs: autonomy, competence and relatedness. Autonomy refers to the sense of choice and control over one's actions, whilst competence involves feeling effective and capable when performing tasks, and relatedness encompasses establishing meaningful connections with others. When these needs are supported, individuals are more likely to internalize and sustain active behaviours. Thus, the application of SDT to PA promotion helps to explain the way in which personal motivations are shaped by need satisfaction (6).

The new materialist approach offers valuable insights for understanding the factors that influence PA practices by emphasizing the entanglement of human and non-human elements. This perspective moves beyond individual agency to consider the way in which environments, objects and social structures interact to shape behaviours. In terms of PA promotion, adopting this lens helps to explore the way in which material elements such as facilities, equipment and, even, natural landscapes co-construct opportunities and barriers to promote or discourage active lifestyles. By looking more deeply at these complex interdependencies, future interventions could be designed which create environments that are more responsive and supportive of PA (7).

Qualitative research methodologies stand out due to their potential to perform a more in-depth examination of the subjective perspective of individuals and complex social processes, alongside their potential for favouring reflective attitudes in participants (8). Within the framework of qualitative research, visual methods may help to foster reflection by favouring the emergence of new ideas and exploring new possibilities to communicate and disseminate knowledge (9, 10). This qualitative approach can be of special interest to get a deep understanding of the complex interplay of different factors influencing PA.

The present qualitative study applied visual methods and called on a sample of research experts in PA promotion. The aim of the research was to identify, from expert opinion, some basic principles to guide PA promotion interventions in society. Gathered information could be useful for improving the design and implementation of future interventions designed to promote PA.

## 2 Method

### 2.1 Participants

The sample included a total of 50 internationally acclaimed researchers, who had participated in research projects and scientific publications related with PA promotion for health over the last decade. Given that participants were selected by the study research team (located in Spain) based on their condition of being “researchers of acknowledged international prestige”, as determined by their peers [due to their long professional careers in PA promotion (>10 years), high publication output and telling participation in PA promotion research projects], the sample could be described as an international sample. Snowball sampling was also employed whereby initially contacted researchers were asked to act as mediators and reach out to other eligible participants. All participants were aged between 31 and 73 (M = 46.24; SD = 9.62) years. Further sample characteristics are presented in Table 1.

**Table 1:** Sample characteristics.

Variables	Frequency (%)	
Gender	Male	23 (46%)
	Female	27 (54%)
Research area	Social sciences	43 (86%)
	Natural sciences	7 (14%)
Country of origin	Spain	26 (52%)
	France	6 (12%)
	United Kingdom	4 (8%)
	Australia	3 (6%)
	New Zealand	3 (6%)
	Ireland	2 (4%)
	Holland	1 (2%)
	Portugal	1 (2%)
	Belgium	1 (2%)
	Germany	1 (2%)
	USA	1 (2%)
	Canada	1 (2%)

### 2.2 Procedure and data collection

The present study received the approval of the Research Ethics Committee of the University of Zaragoza [Research Ethics Committee of the Autonomous Community of Aragon (CEICA) with the ethics reference number: C1P117/0018]. Following review of study information pertaining to study objectives and procedure, participants provided informed consent online and stated their official contact information.

All study data were obtained via an online questionnaire (i.e., Google form), which was emailed personally to each researcher, followed by up to two reminder emails over a three-week period to encourage participation. This questionnaire, in addition to compiling data on participant characteristics (gender, research area and country of origin), gathered study data as outlined below. Only three international experts did not return the online questionnaire after being sent the two reminders.

Firstly, an image bank of 14 images selected by the research team was presented to the participants. All images were related to different common PA engagement scenarios. Next, participants were asked to choose one, two or three photos which stood out to them and, in their opinion, could be used to represent a basic principle of PA promotion that was of importance to them. For each selected photo, participants were requested to suggest a title (“Photo title”) and explain the basic principle for PA promotion (“Explanation of the basic principle”). The “Photo title” could be a word or short sentence that reflected the basic principle represented by the photo. In the case of a full sentence, the statement could be original or reflect a famous quote from a reputed author. With regards to “Explanation of the basic principle”, participants were requested to describe what they believed to be the basic principle for PA promotion in greater detail.

Given the possibility that participants considered basic principles that they did not believe to be related to any of the preselected 14 images presented, they were also given the opportunity to submit a maximum of three images which were not contained by the initial set of 14 images. As was the case above, such images were also submitted with a title and explanation of the basic principle. The only condition was that all images had to be copyright-free.

#### 2.2.1 Development of the image bank

In order to facilitate the photo-elicitation process, a set of 14 images was carefully developed and curated to depict a broad range of common PA engagement scenarios. The selection process followed a structured procedure based on both theoretical and practical criteria.

First, the research team conducted an internal brainstorming session informed by the ecological and salutogenic frameworks underpinning the study. This process generated a list of potential PA settings and behaviors that should be represented in the image bank, covering individual, social, and environmental contexts (e.g., active transportation, play, sports participation, outdoor recreation, gender diversity, and different age groups). Second, a pool of candidate images was sourced from publicly available, copyright-free photographic databases, as well as from previous project documentation and the personal images belonging to some researchers. These candidate images were evaluated based on diversity, contextual richness, clarity, and their potential to elicit reflection and dialogue among experts. Third, a panel of five members of the research team independently rated each image based on its representativeness and alignment with the study objectives. Images were selected to maximize variation across dimensions such as setting (e.g., urban/rural), age group, gender representation, type of activity (e.g., organized vs. spontaneous), and social configurations (e.g., individual vs. group activity). Finally, the 14 images with the highest composite scores were selected as the final stimuli. These images were then reviewed in a final consensus meeting to ensure content validity and to avoid overrepresentation of some considered dimensions.

This rigorous procedure aimed to reduce selection bias and ensure that the visual stimuli provided a rich, inclusive, and balanced foundation for eliciting expert perspectives on guiding principles for PA promotion.

### 2.3 Data analysis

Qualitative data gathered in the present study was analysed with the aid of NVivo software, which was used to efficiently organise and classify all information (11). Data were analysed by means of thematic analysis (12). First, written text was read and a general review of submitted images was performed to acquire a global idea of existing information. Second, the fragments of text that represented basic PA promotion principles were coded, together with the images that they were associated with. This initial coding process was both inductive and descriptive. Later, a deductive process was used to searching for themes, and the researchers considered the socio-ecological framework to be useful for conceptualising the main themes (13, 14). In line with this, generated codes were classified into the following themes: “PA promotion principles related to personal factors”; “PA promotion principles related to the social environment”, and; “PA promotion principles related to the physical environment”. A fourth theme was conceived that was entitled “Global PA promotion principles”. This theme encapsulated codes which reflected cross-cutting issues that did not clearly match any of the three previous themes but were of great importance. Sub-themes for each of the main themes were defined inductively and descriptively.

The person responsible for leading the analysis, VB, drafted different thematic maps to graphically present the results of the analysis to the other four members of the research group (EG, JJ, JZ, LG) who played the role of critical friends (15). At a meeting, VB presented the thematic maps, as well as the codes included within each theme/sub-theme, and answered the critical friends' questions and suggestions. Finally, the research group conceived a system of themes and sub-themes which provided a structure for the redaction of findings. The incorporation of critical friends during the analytical process helped to improve the rigour and credibility of data analysis (15).

## 3 Results

The findings of the present study are divided into four sections: 3.1. Global PA promotion principles; 3.2. PA promotion principles related to personal factors; 3.3. PA promotion principles related to the social environment; 3.4. PA promotion principles related to the physical environment.

Each section describes information provided by participating experts. Some of the original expert responses are cited throughout the results section in cases in which they are especially revealing. The images deemed by experts to be best related to their written statements are also exhibited as figures throughout the manuscript. Images initially included in the questionnaire or provided later by the experts themselves are differentiated to facilitate better understanding of this section.

The number of experts to support a certain idea is described throughout the results, although it should be acknowledged that the ideas generated through the thematic analysis are more important than the number of experts to support each idea. In other words, an idea could be mentioned by only one expert that might be just as relevant as those referred to by a large group of experts. In this way, the thematic analysis conducted in the present study allows for these relevant “single” ideas to also be highlighted.

### 3.1 Global PA promotion principles

One participating expert indicated that, in order to achieve true PA promotion, multicomponent interventions must be considered. Such interventions should empower all community members and affect different social contexts and populations.

Two experts (one man and one woman) highlighted that scientific evidence must guide PA promotion interventions, as it may provide key knowledge regarding target populations and intervention contexts. Emphasizing the need for community-centric approaches, one expert mentioned:

I believe it is important to stop talking about people and start talking to people, giving them agency (capacity to act) and skills to make the decisions they want to make, to live the life they decide to live (expert 21, female).

Another female expert emphasised that it is of great interest to build social awareness about public health. It is important for the population to understand that engaging in PA, in addition to producing personal benefits, will produce social benefits in terms of public health and quality of life, with the latter reflecting an aspect that will give rise to a better society.

**Figure 1:**
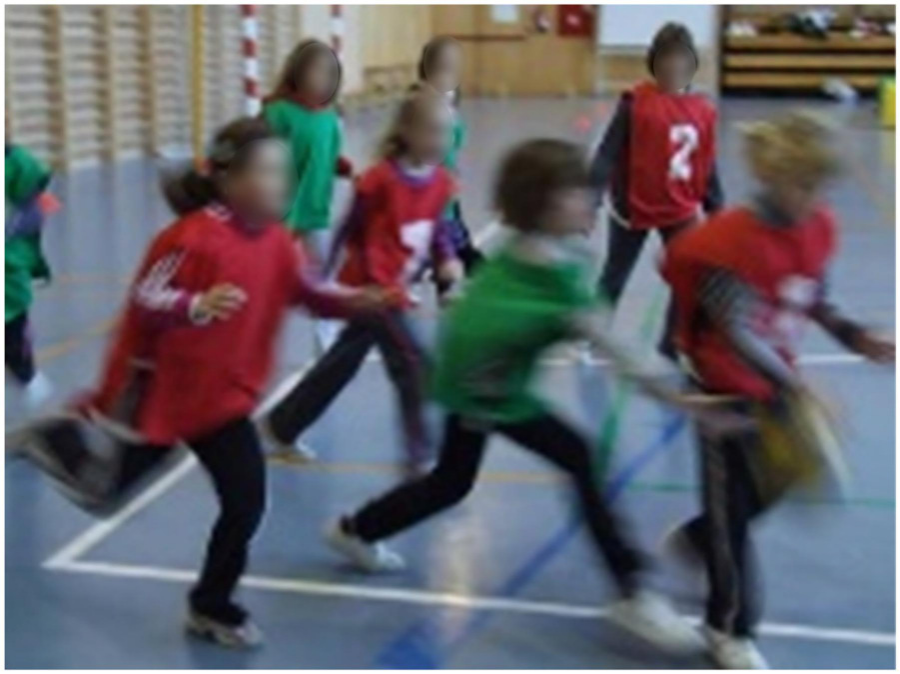
Image 10 of the online questionnaire.

One male expert from the field of Natural Sciences recalled that it is necessary to promote the right intensity of PA to produce biological health benefits. In this way, sport and warmup games may provide suitable resources (see Figure 1).

**Figure 2:**
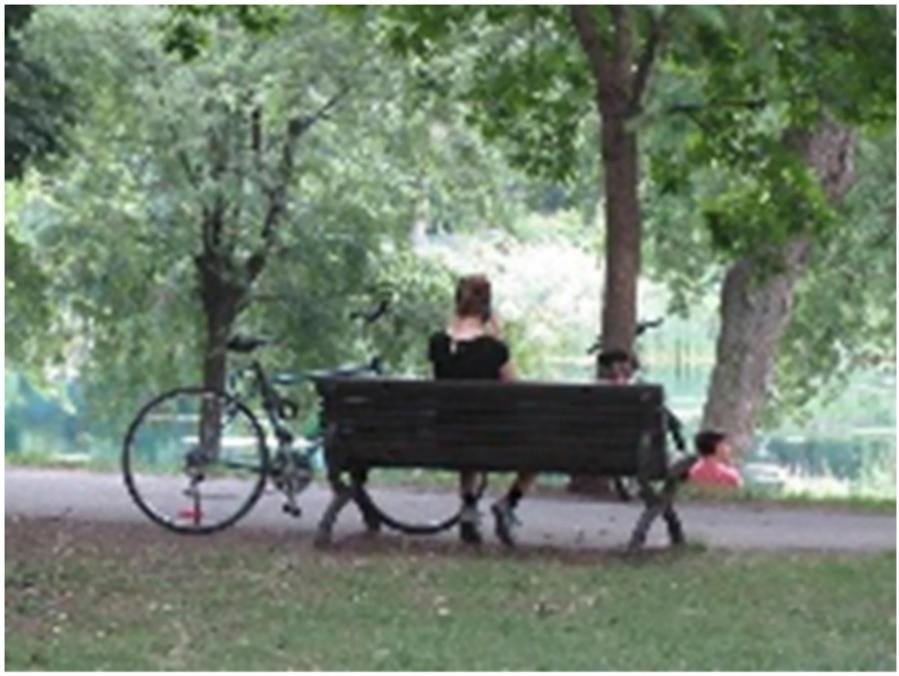
Image 8 of the online questionnaire.

However, one social sciences expert made the following reflection:

Antonovsky's salutogenic approach to health promotion places the concept of Sense of Coherence at the heart of health maintenance. Too much attention in terms of health promotion that seeks to promote a physically active lifestyle focuses on risk; the risk of cardio-vascular disease, the risk of obesity. Such an approach often promotes moderate to vigorous PA as the answer to a range of health-related issues. A salutogenic approach, in contrast, is concerned with the salutary factors that are implicated when it comes to people staying healthy. The capacity to make sense of one's life, whilst also viewing life as being manageable, meaningful and comprehensible is, as Antonovsky suggests, of fundamental importance to health and wellbeing. This photo very nicely captures two salutary factors, namely, activity (in the form of cycling) and contemplation (taking a quiet moment to yourself in a pleasant environment) (expert 40, male; see Figure 2).

In concordance with this expert, another three experts (men) from the field of Social Sciences cautioned that it is important not to focus exclusively on the quantitative components of PA (frequencies, duration, intensity, etc.), but also on the qualitative aspects of PA (social inclusion, ethical considerations, positive subjective experiences of PA engagement, expression of creativity and art through PA, making PA a lifestyle priority, and other important habits related with health and wellbeing, etc.).

### 3.2 PA promotion principles related to personal factors

Four experts (three women and one man) mentioned the importance of favouring the learning of motor skills in order to enable individuals to engage in PA. Further, it is necessary for people to believe that they possess sufficient knowledge, as well as the appropriate cognitive, emotional and motor capacities needed to be physically active (e.g., to engage in swimming). However, it is also important for people with fewer motor skills and lower physical fitness to experience success in physical-sporting contexts and, consequently, feel that they are competent when it comes to motor skills.

**Figure 3:**
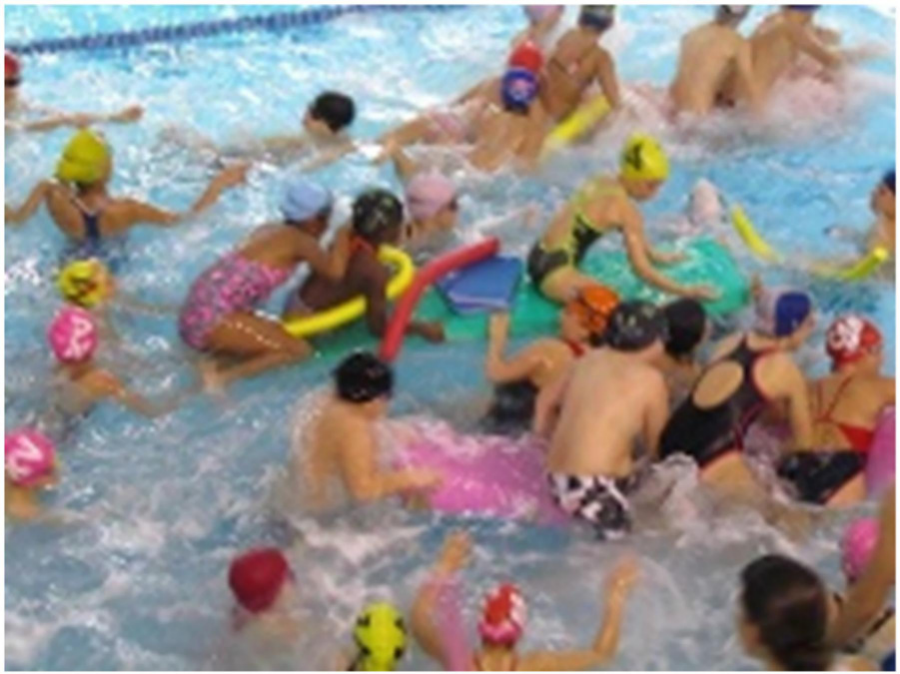
Image 1 of the online questionnaire.

Notably, 21 experts (12 men and 9 women) mentioned that it is highly important that experiences of PA are enjoyable as a means of encouraging continued engagement. Together with the term “fun”, the experts referred to the need for enjoyment, happiness, pleasure, play and positive experiences when engaging in physical-sporting activities (see Figure 3).

Four experts (men) considered it necessary for people to show commitment, determination and motivation towards maintaining an active lifestyle. In terms of motivation, two of these experts referred to the SDT (16) and, with it, the need to satisfy the basic psychological needs of competence, autonomy and relatedness as a means of supporting people to foster self-determined motivation and, consequently, continue to engage in PA over the long term.

**Figure 4:**
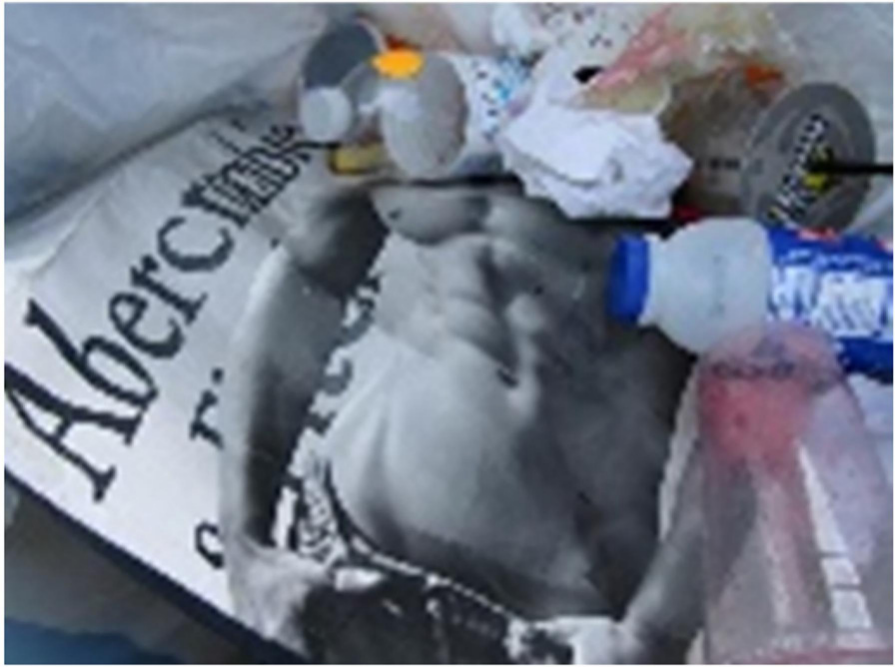
Image 4 of the online questionnaire.

Three experts (two women and one man) remarked that individuals must be trained to be critical thinkers who are able to make appropriate decisions for their health and wellbeing within the landscape of today's complex society. One female expert recalled that the population must also be aware of the risks of PA. Another female expert referred to the “body cult” fostered by present society, which is based on “normative models of femininity and masculinity that determine what is defined as a healthy body in a very restrictive manner (muscular for men; slim and toned for women)”. Initiatives are required to tackle this conception, as it leads individuals to engage in PA as a means of improving their body shape and obtaining the “ideal body” rather than to improve their health and wellbeing. The male expert supported this view, as reflected through the following statement:

Encouraging people to make healthy lifestyle choices in an environment that is increasingly unsupportive of such choices will require us to come up with smart approaches that consider, among other things, the powerful economic and market forces that are shaping our lifestyles in modern societies (expert 36, male; see Figure 4).

### 3.3 PA promotion principles related to the social environment

#### 3.3.1 Long-term collaboration between people and organisations

**Figure 5:**
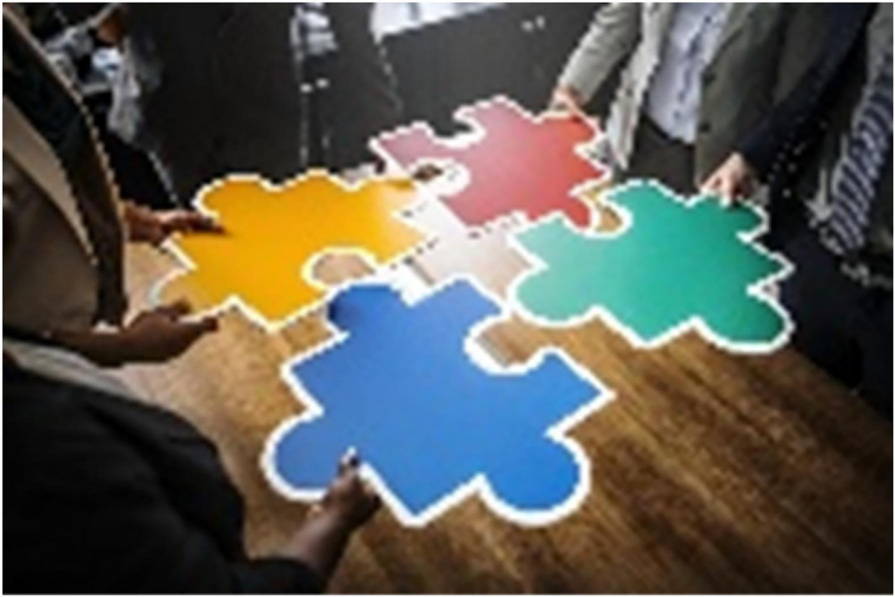
Image provided by participant 22 (female).

Two experts (one woman and one man) highlighted that the social promotion of PA requires long-term collaboration between people and organisations from different sectors. Such collaboration should be based on mutual trust between the different agents involved (see Figure 5).

#### 3.3.2 Promoting PA in all populations with the inclusion of all sectors

**Figure 6:**
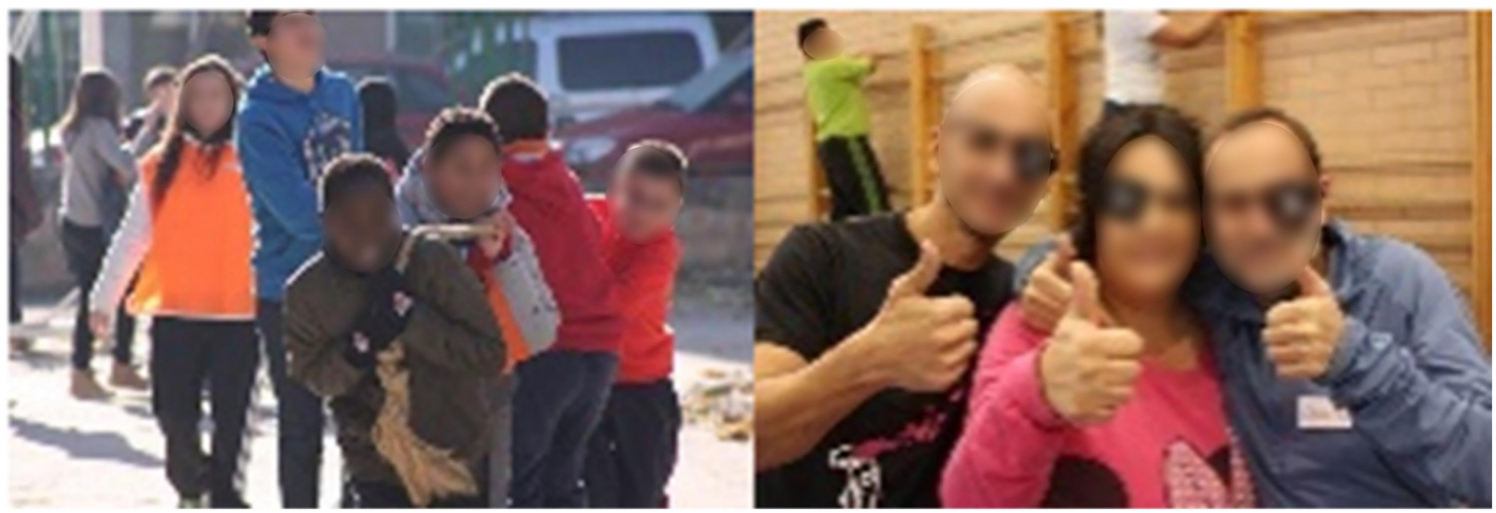
Image 5 and 14 of the online questionnaire.

One female expert made a very poignant thought when outlining important factors to be considered when developing interventions aimed at promoting PA:

Interventions run the risk of increasing social health inequalities if they fail to consider the needs of different population groups as they will only or mainly reach more privileged groups, shutting out vulnerable groups, which are actually the groups that could most benefit from such interventions (social class, ethnic group, gender, etc.) (expert 31, female; see Figure 6).

In this sense, four experts (three women and one man) suggested that approaches to PA promotion should include strategies targeted towards special, vulnerable or underprivileged populations (people with disability, people from minority ethnic groups, obese individuals, individuals with low socioeconomic status, the elderly, etc.). These population groups typically present high levels of physical inactivity and face a lack of opportunities for PA engagement. Two experts urged the need to offer specific PA programmes for such population groups, as a potential means of making them feel more comfortable and supporting them to feel more supported and understood (see Figure 6):

Scientific evidence has begun to acknowledge the effectiveness of PA interventions designed for groups with special characteristics. Thus, an obese person will feel more comfortable within a programme or intervention designed for obese people, as they will feel identified with that group of people (expert 16, female).

PA is important for people with disabilities and, given that disability may be a barrier to exercise, it is important that adapted programs exist with the supervision of trained professionals. People with disabilities or illness are often more motivated to engage within a group of their peers because they feel better understood and more supported (expert 35, female).

**Figure 7:**
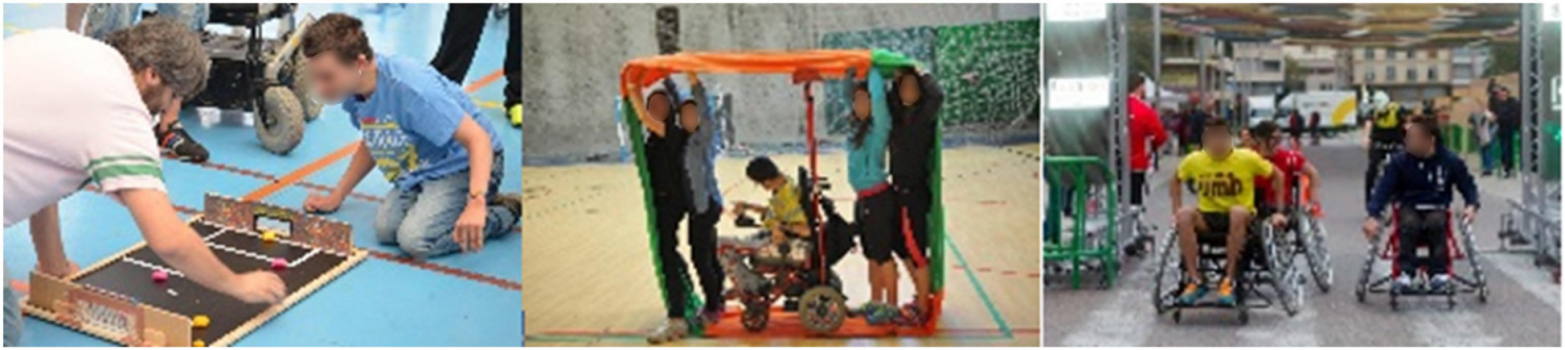
Images provided by expert 13 (male).

Another male expert (13) emphasised the need to also promote activities that offer people from vulnerable groups the chance to integrate within the wider general population, thereby favouring their genuine inclusion in society. Such activities or programmes should incorporate activities that favour equitable participation (see Figure 7).

This same expert (13) suggested that it would be useful to capitalise on certain sporting events to give greater visibility to and normalise the involvement of individuals with disabilities. Such an approach would favour the genuine inclusion of this group in society and increase their participation in PA and sport contexts:

This photo is an exhibition of a wheelchair race performed within the context of a fun-marathon (sporting activities and races for children and young people) in the build to the Elche half marathon (Elche, Spain). The image presents a PA and Adapted Sports lecturer from Miguel Hernandez University and two of their students (one who has a disability and is a regular wheelchair user) crossing the finish line. The image reflects the high degree of complicity achieved due to the inclusive strategies carried out during the activity, as witnessed at the time by the dozens of people present in the crowd (expert 13, male; see Figure 7).

**Figure 8:**
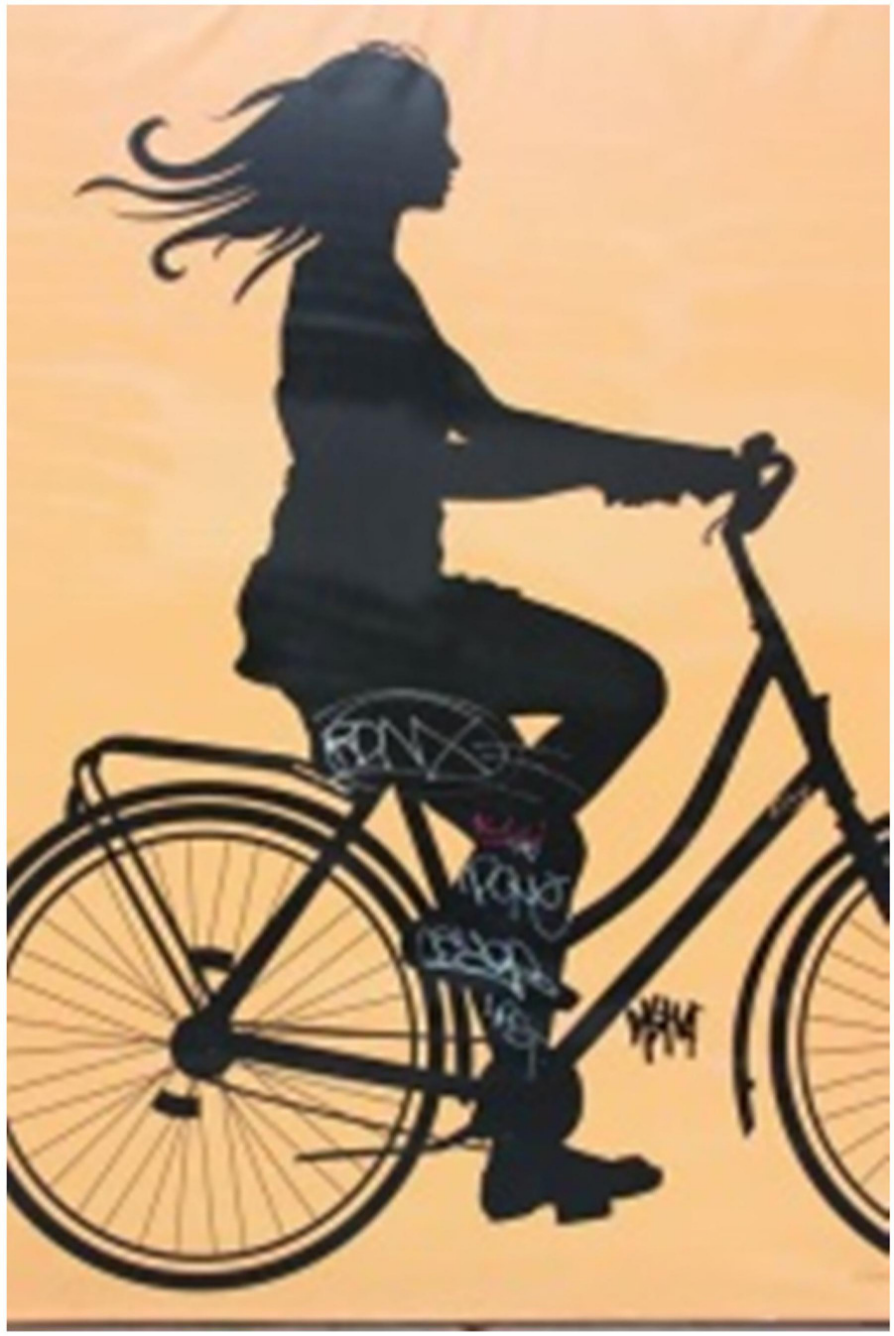
Image 12 of the online questionnaire.

Six experts (four women and two men) outlined the need to take a gender perspective when designing PA promotion strategies. According to these experts, females should be specifically targeted, given the fact that they tend to engage in less PA and that traditional PA provision does not cater to their interests. For example, at early ages, opportunities to participate in competitive sport are usually less attractive to females. Thus, PA must be promoted both in Physical Education classes and in society in general, whilst equally respecting the needs and interests of both boys and girls. Joint participation at schools should be prolonged for as long as possible and favour activities that can be experienced by both males and females together going into and throughout adult life (see Figure 8).

Women need to be encouraged to build PA into their daily lives and to realise that this does not need to involve competitive sport (expert 26, female).

One of the basic principles of PA promotion should be based on activities that are co-experienced by males and females, not only, in school sport, but, also, at all life stages (expert 8, female).

#### 3.3.3 Favouring inclusive social contexts for PA engagement

**Figure 9:**
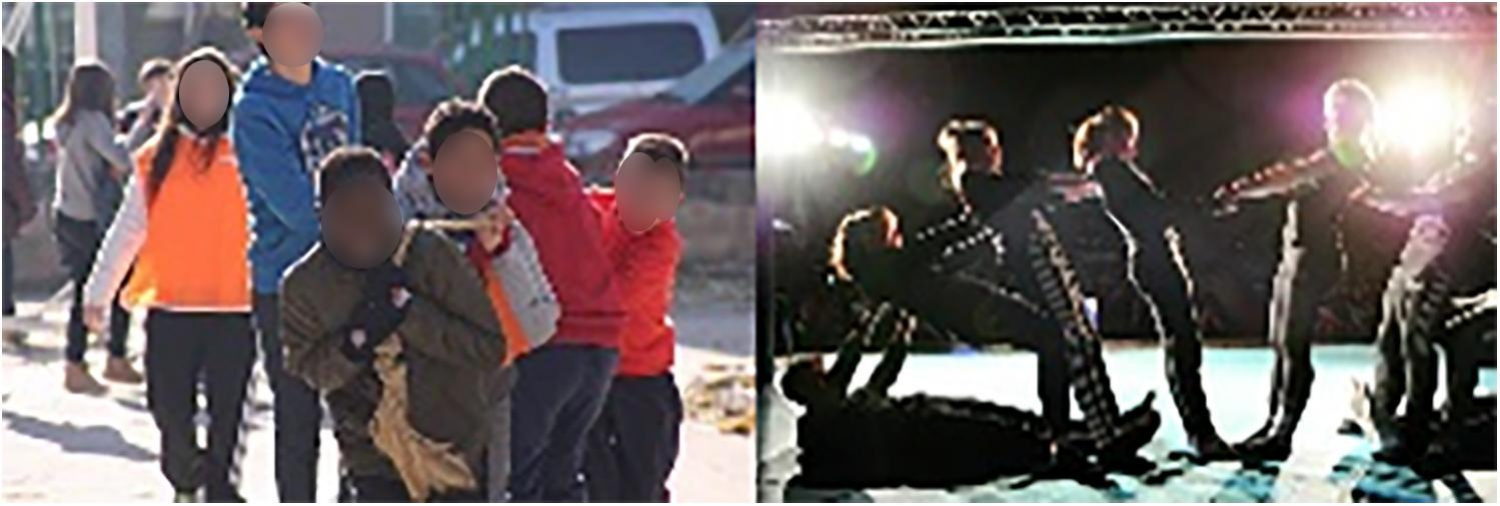
Image 5 and 13 of the online questionnaire.

It serves to note that 17 experts (10 women and 7 men) highlighted the importance of favouring inclusive PA contexts as a means of promoting active lifestyle habits in society. This is crucial, not only for groups with special needs, but, also, for the wider population. In addition to the term “inclusion”, experts referred to socially favourable contexts, in which individuals cooperate and, therefore, feel social support and believe themselves to form an important part of society. In such contexts, diversity is tolerated, and prejudice and stigmatisation towards others is avoided (see Figure 9).

PA should be fun and something that you learn to do with friends at school. PA must be socially safe, where participants feel that they can take part without fear of being stigmatised if they are not technically or physically competent (expert 43, male).

Activities that we perform with others are particularly beneficial for our mental health. We feel like we are part of a team, we develop bonds with others, we feel accountable; therefore, we are more likely to attend subsequent training sessions or games (expert 28, female).

#### 3.3.4 Extracurricular sport provision and PA programmes

**Figure 10:**
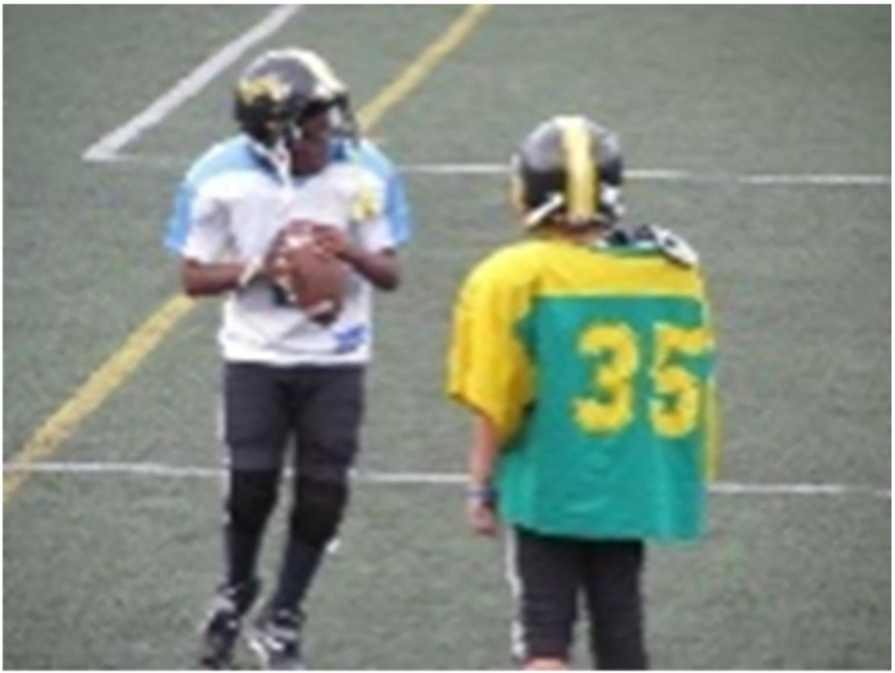
Image and 6 of the online questionnaire.

Three experts (men) indicated that, beyond school Physical Education, which will be discussed later in this article, extracurricular PA and sport programmes can be very useful for promoting PA during the first stages of life (see Figures 3 and 10).

#### 3.3.5 The role of key social agents with regards to PA promotion

##### 3.3.5.1 The media and advertising

**Figure 11:**
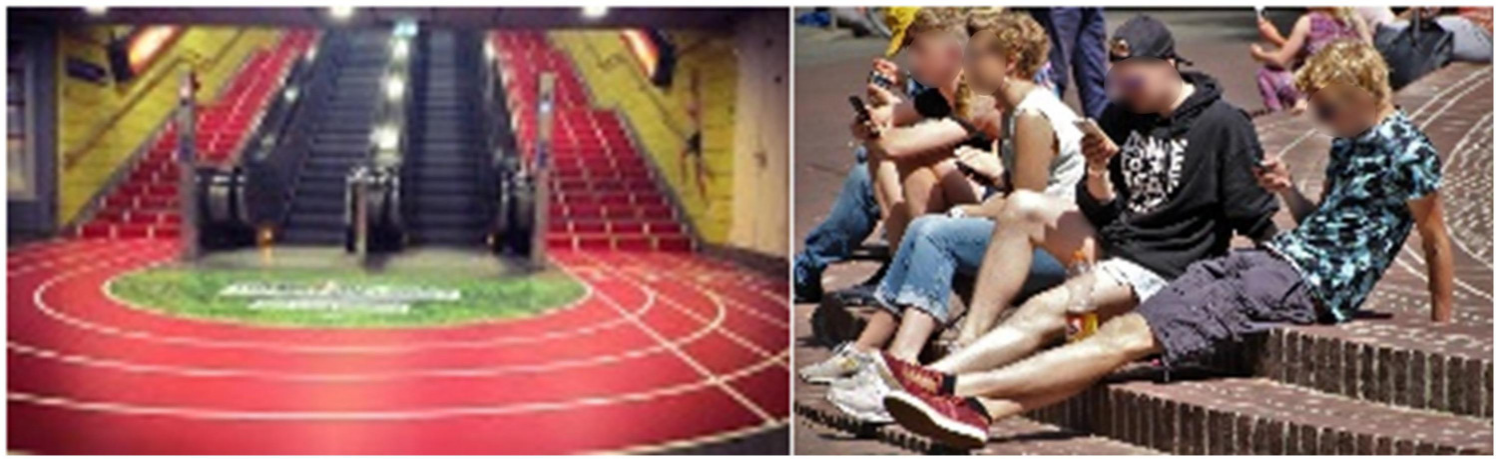
Images provided by expert 34 (male) and expert 46 (male).

One male expert (34) referred to the influence of the Media and advertising with regards to PA promotion. He highlighted that advertisements displayed through the television and other media, in addition to marketing products, can also enhance the acquisition of healthy habits such as PA engagement. This participant provided the following image from an advertising campaign (see Figure 11):

Another male expert (46) indicated that new technologies (mobiles, tablets, wearables, etc.) are going to be crucial for PA promotion and the prevention of physical inactivity (see Figure 11).

##### 3.3.5.2 The family

**Figure 12:**
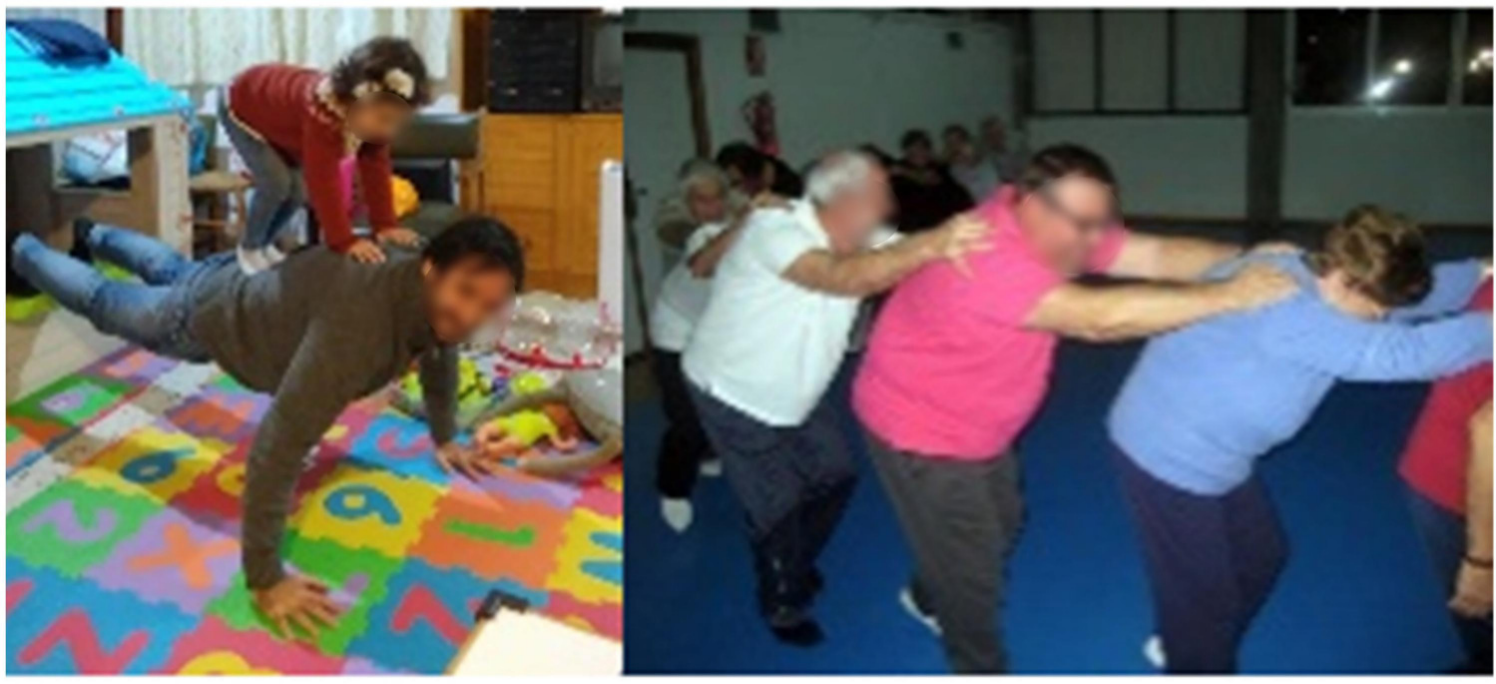
Images provided by expert 12 (male).

Seven experts (4 men and 3 women) commented that parents play an essential role in promoting the PA of their children. Parents must serve as role models and lead the way to inspire their children to be physically active (see Figure 12).

Leading by example isn't another way to teach, it is the only way to teach (Quote from Albert Einstein, mentioned by expert 12, male).

In addition to engaging in PA themselves and setting an example for their children, parents should also engage in exercise with them and support them to engage in PA and sport autonomously. The family provides the bedrock of education and joint PA engagement can help to strengthen family ties.

Finally, in addition to parents, siblings and grandparents can also play a very important role in promoting children's PA engagement. Moreover, it is important not to forget that children can also have a reciprocal positive influence on making their parents and grandparents active.

##### 3.3.5.3 The school

Twelve experts (eight men and four women) referred to school Physical Education classes as one of the main means of promoting PA in society (see Figure 1):

The most important paths through which to promote physical exercise, after the family, is the education system (expert 12, male).

The school guarantees that PA experiences can be lived by all, regardless of personal or family contexts. The school is obliged to extend the benefit of education, and, by extension, of physical education to all children. It is the place to break down barriers (personal, social, etc.) and to ensure that all children have good PA “experiences” (expert 49, male).

These experts highlighted the importance of training Physical Education teachers by equipping them with the necessary didactic resources to favour student motivation towards PA and ensure positive experiences when doing the subject. They also emphasised the importance of meaningful learning and the need to involve students in PA and make it a key part of their lives at an early age.

Tell me and I forget, teach me and I may remember, involve me and I learn (quote from Benjamin Franklin, mentioned by expert 12, male).

Experts warned of the importance of favouring learning and motor skill development and fully including students with lower motor skills and worse physical fitness. In this sense, experts urged the need to adapt the rules of games and competitions to favour inclusion. For example, a useful approach could be to organise sports competitions that award prizes, not just to the winners, but, also, to those who stand out by exhibiting values associated with sport, such as participation, effort, personal growth and respect.

Experts also considered that it is necessary to increase the time dedicated towards Physical Education classes. To this end, it was suggested that interdisciplinary projects should be introduced to extend active learning from Physical Education to other subjects such as Maths or Geography.

Finally, experts indicated that, beyond Physical Education, school represents a privileged context in which to develop PA promotion interventions, with the involvement of students, teachers and families. It is essential that such interventions link the school setting with other relevant contexts in terms of leisure as a means of transferring learning and vicarious experiences lived in Physical Education to other facets of life. In this regard, experts argued that educational and health policies should be coordinated to increase effectiveness.

##### 3.3.5.4 PA and sport professionals

Six experts (four men and two women) expressly mentioned that it is necessary professionals in the field of PA and Sport to be well-trained to achieve real social promotion of PA. Such professionals must be competent and self-demanding. They must also be kind, respectful and thoughtful, above all when working with vulnerable populations and those with special needs. Humour is also considered to be a key methodological resource for maximising the benefits of PA programmes for populations with different illnesses (see Figure 12):

A positive climate must be fostered in sessions, using humour and distracting participants from focusing on their problems or pains (expert 12, male).

##### 3.3.5.5 Peers

Two experts (one woman and one man) stated that the role of peers is essential for promoting PA and preventing sedentary behaviour:

Peers are determining factors of engagement and, in contrast, of inactivity (expert 14, male).

The influence of friends is key when making the transition from childhood to adolescence. Thus, it is necessary to promote physical activities and environments of a social nature, where young boys and girls can move and enjoy moving in the company of young people of the same age (expert 29, female).

##### 3.3.5.6 Caregivers

One male expert (12), who has been charged for more than 7 years with the design, start-up and evaluation of a PA programme for people with mild cognitive impairment and mild Alzheimer's, emphasised the key role of caregivers when it comes to promoting PA in dependent populations:

In the case of people with a certain degree of dependence, it is necessary to understand the importance of involving the relatives/caregivers who are responsible for them, as they are the ones who encourage them and take them (expert 12, male).

### 3.4 PA promotion principles related to the physical environment

#### 3.4.1 The provision of suitable physical environments for PA engagement

Another point worth discussing is that 20 experts (12 women and 8 men) indicated that, for PA promotion at a societal level, it is necessary to provide people with natural, green, pleasant, clean, pedestrian and safe environments, which support the maintenance of active lifestyles. Experts referred to natural spaces which can be located both outside of cities or integrated within them, such as is the case with pedestrian zones, parks, etc.

It is important to highlight that participating experts placed a high degree of importance on two main aspects. The first aspect was that environments need to be pleasant (e.g., natural, green and pretty spaces). The second aspect was that natural spaces must be safe from both a physical (e.g., traffic free spaces or areas separated from cars to avoid accidents) and social viewpoint (e.g., low-crime areas).

**Figure 13:**
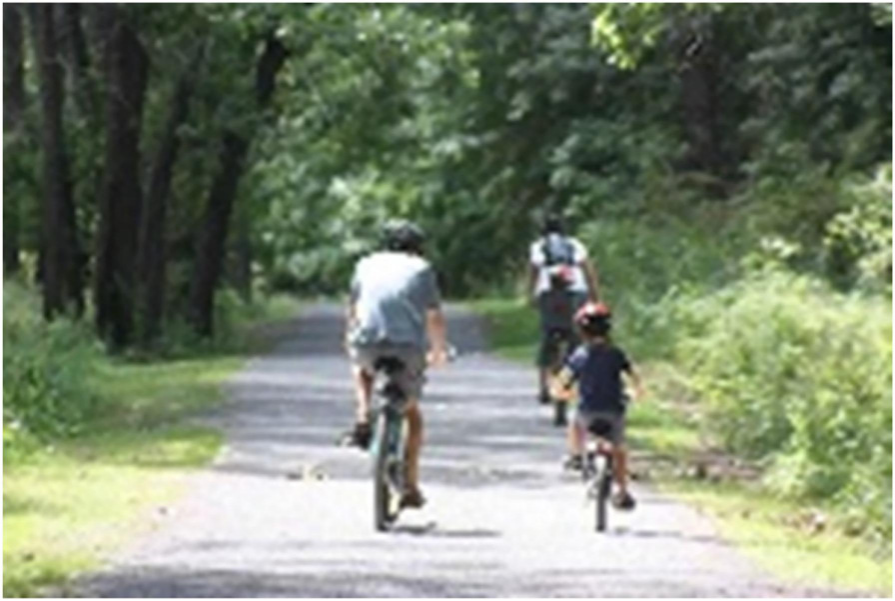
Image 9 of the online questionnaire.

Environments that move us. The adoption of PA behaviour cannot be considered as being only dependent on an individual decision. The “environments that move us” principle refers to the many different interpersonal, organisational and environmental factors that act as facilitators or barriers to the adoption of PA behaviour. This photograph particularly illustrates the impact of social influence (family) and having an appropriate physical environment to perform PA safely (expert 29, female, see Figure 13).

**Figure 14:**
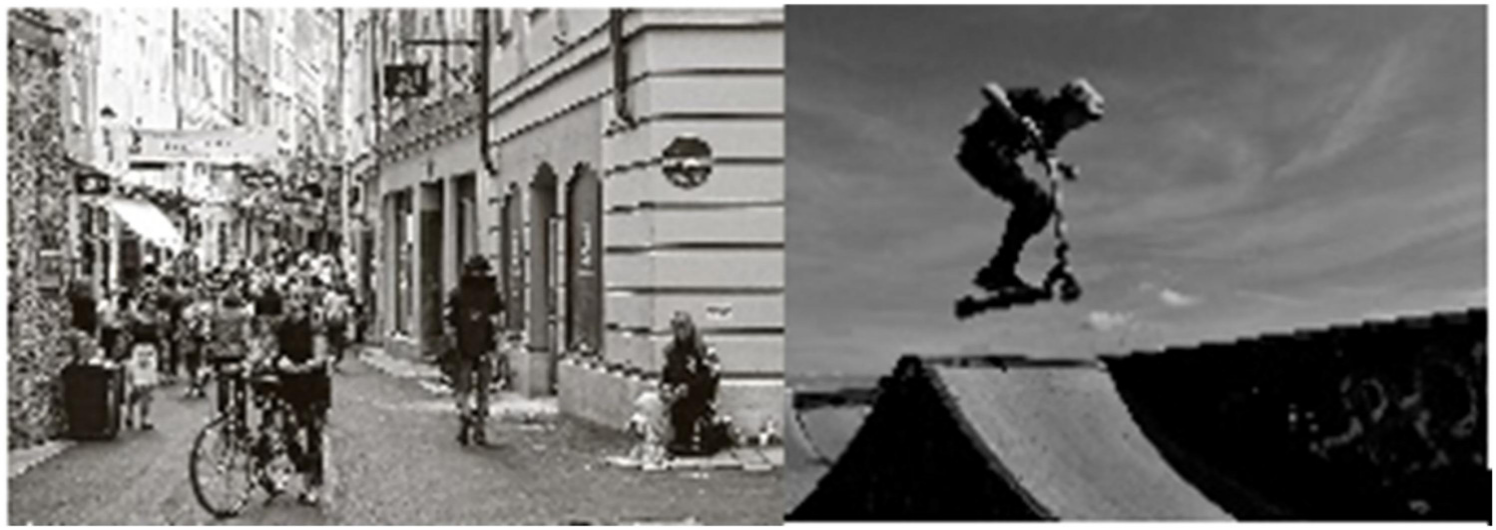
Image provided by expert 30 (male) and 43 (male).

The selected image exhibits many people who are moving actively (i.e., walking) along a city street. In my opinion, this photograph perfectly exemplifies the importance of urban environments in promoting PA in the population. More specifically, I am referring to urban environments that are designed to give priority to walking or cycling, rather than using private motorised transport, which would be the case of spaces with cars (expert 30, male, see Figure 14).

Experts also highlighted that the availability of pleasant and safe environments in which to engage in PA would bring about other benefits beyond the improvement of health and wellbeing due to actual PA engagement. It was stated that merely spending time in natural environments can have a positive influence on mental health, psychological wellbeing and stress reduction, and lead to the experience of pleasant and stimulating sensations.

Further, pleasant and safe environments in which to engage in PA may favour free play in children, something that is essential in current society:

With the increased focus on highly organized and structured PA programs, opportunities for children to engage in free play are lacking, which reduces spontaneity, initiative, and enjoyment. At the same time, the availability of appropriate places, spaces and facilities with appropriate supervision may facilitate free play, even as a break in the context of organized programs (expert 36, male).

Too often young people are encouraged to be active via particular sports or pastimes. We often forget or ignore some of the things they would choose if given a choice. We must celebrate and encourage the choices young people make and encourage them to be active in whatever ways suit them (expert 43, male; see Figure 14).

According to participating experts, pedestrian and safe spaces in urban areas can also strengthen social ties between the population and small business by shaping economic activity. Finally, moulding urban environments that encourage people to travel by bike or on foot will help to reduce motorised traffic and resultant pollution. This type of urbanism and active lifestyles in green and pleasant environments can contribute to raised environmental awareness within the population. It is also important to note that active transport is economically more efficient than motorised transport (see Figure 2).

#### 3.4.2 Active transport promotion via adequate resources and equipment

**Figure 15:**
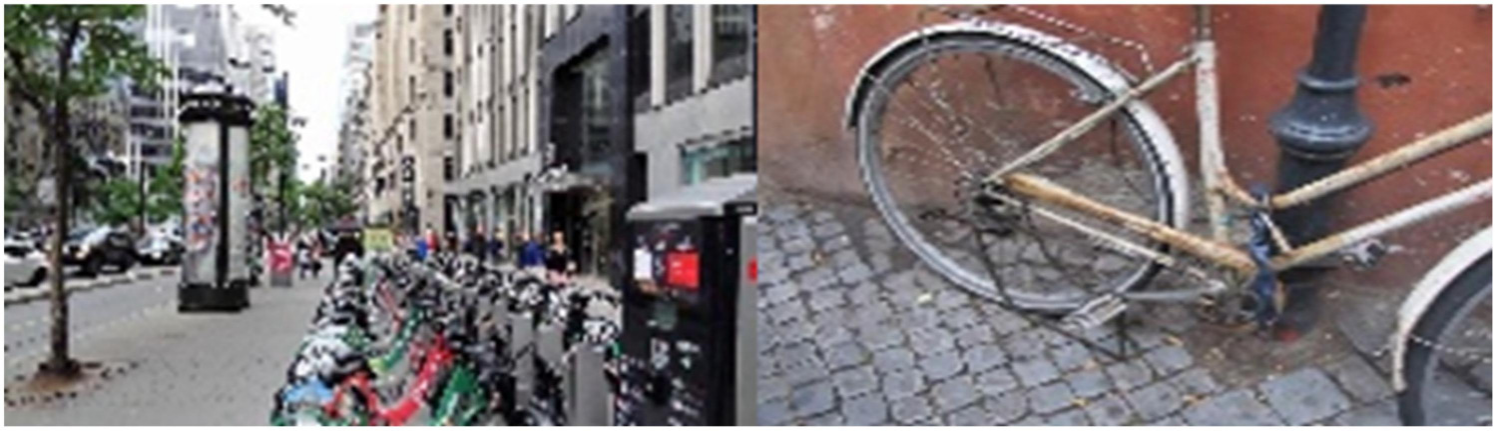
Image provided by expert 32 (female) and image 2 of the online questionnaire.

Ten experts (6 women and 4 men) expressed the need to favour active transport as a means of PA promotion. To this end, the population must have access to safe and quality equipment. In this sense, study experts outlined several strategies, such as installing public bicycle schemes in cities and offering bicycle repair/inspection services, parking areas and padlocks so that bicycles can safely be left unattended, as well as providing showers, irons and ironing boards for those who cycle to work. Experts also urged the need to promote active transport to school by ensuring safe cycling paths in both a social and physical sense (see Figures 13 and 15).

## 4 Discussion

The present study offers valuable insights into the guiding principles of PA promotion from the perspective of internationally recognized experts. Present findings underscore the importance of adopting a multilevel and multicomponent approach by integrating individual, social and environmental factors when promoting active lifestyles. Findings align with the socio-ecological model (14, 17), which emphasizes that effective PA promotion requires addressing, not only, individual behaviors, but, also, the social and environmental contexts in which they occur.

From a global PA promotion principles perspective, an important finding emerging from the present study was the subtle yet meaningful tension between experts with backgrounds in the natural sciences and those in the social sciences. While the former often emphasized the quantification of PA, especially in terms of frequency, duration, and intensity, those from social sciences stressed the qualitative dimensions of the experience, such as enjoyment, meaning, autonomy, and context. This divergence reflects deeper epistemological orientations, with objectivist and biomedical paradigms emerging on the one hand, vs. constructivist and socio-cultural paradigms, on the other. This illustrates the way in which disciplinary backgrounds can shape, not only, the interpretation of what “effective promotion” entails, but, also, the types of strategies considered to be valuable. The recognition and integration of these differing perspectives could strengthen future PA promotion efforts, especially when designing multicomponent and context-sensitive interventions. This interdisciplinary tension should not be viewed as a limitation but rather as an opportunity for epistemological enrichment, leading to the design of more holistic and impactful intervention programs.

At an individual level, psychological factors such as motivation, enjoyment and motor competence clearly emerge. Experts highlighted the importance of satisfying basic psychological needs, as outlined in self-determination theory (16), as a means of fostering long-term adherence to active behaviors. This suggests that interventions should not solely focus on promoting PA for health outcomes, but also aim to create enjoyable and intrinsically motivating experiences that reinforce a sense of autonomy, competence and social connectedness (18). These findings resonate with the SDT (16), especially through their emphasis on autonomy, competence, and relatedness as drivers of sustained engagement. Several experts, particularly those from a social science background, explicitly articulated the need to create contexts that nurture these basic psychological needs. However, the emphasis on measurable outcomes from other experts may clash with SDT's call for more person-centered and process-focused approaches, indicating a possible misalignment in the way in which success is defined in terms of PA promotion. Considering the background of stakeholders, practitioners and those responsible for PA intervention programs seems to be of paramount importance for correctly tackling PA promotion.

Social factors were also identified as being critical for PA promotion. Experts pointed to the key role of families, peers, educators and the Media in modeling and supporting active behaviors. In line with previous research (2), fostering inclusive and supportive social environments may help overcome barriers to PA, particularly in vulnerable groups. Moreover, attention to gender perspectives and the specific needs of marginalized populations remains a crucial area for the development of equitable interventions (19). An unexpected finding was the relatively limited emphasis on digital environments and their dual role as both barriers and facilitators of PA. While screen time is often problematized, some experts hinted at the potential of digital platforms for enhancing motivation and access. This area may warrant deeper exploration in future studies.

A spotlight was also shone on the physical environment. Consistent with the principles of New Materialism (7), the availability of safe, pleasant and accessible spaces for PA can significantly influence behavior. Experts stressed the importance of designing urban spaces that promote active transportation and facilitate opportunities for spontaneous play and PA across the lifespan (20). Furthermore, perspectives that are aligned with New Materialism, despite being less frequent, challenge traditional human-centered views by advocating for recognition of the way in which material and spatial environments actively shape PA behavior. This was particularly evident in discussions about urban design and the agency of non-human actors (e.g., infrastructure, climate, technology), highlighting the growing influence of post-humanist thinking in health promotion. This approach contributes, not only, to individual health, but, also, to broader societal and environmental benefits.

Overall, findings of the present study provide a comprehensive framework that can be used to guide policymakers, educators and health professionals in designing more effective and sustainable PA promotion strategies. By addressing individual motivations, leveraging social networks and transforming physical environments, interventions can create supportive ecosystems that foster active lifestyles across the lifespan. The integration of these guiding principles will help to ensure that PA promotion moves beyond isolated initiatives and becomes embedded within broader social, educational and political frameworks (21). This holistic approach is particularly relevant in addressing the complex challenges (22) posed by physical inactivity, sedentary behaviour and health inequality, ultimately contributing to the emergence of healthier and more resilient communities. The nuanced insights provided in this manuscript underline the value of combining descriptive and interpretive analysis, revealing how expert knowledge is not monolithic but shaped by multiple positionalities. They also support a move towards more holistic, flexible and context-sensitive strategies for PA promotion, as a means of embracing complexity rather than stifling it.

A key strength of the present study lies in its use of a diverse and internationally recognized sample of experts, which enhances the credibility and relevance of findings. The integration of visual methods also represents an innovative methodological contribution to research on PA promotion. It must be highlighted that the present study also serves to demonstrate the potential of visual methods as powerful tools for eliciting rich and reflective narratives. As highlighted by Glaw et al. (9) and Laholt et al. (10), such methods encourage participants to articulate complex ideas and emotions that might not otherwise surface when using traditional qualitative techniques. The integration of photo-elicitation allowed experts to express abstract concepts through tangible imagery, which enrichened subsequent thematic analysis.

Nonetheless, it is also important to acknowledge certain limitations. The sampling approach was non-probabilistic and may reflect a bias toward Western cultural perspectives (held by the majority of Spanish researchers) and, obviously, personal experiences (experts form Social Science). Whilst diversity was sought in the sample, potential cultural and discipline-specific bias cannot be fully ruled out. This should be considered when interpreting the transferability and generalizability of the findings to other geographical, cultural, or disciplinary contexts. Additionally, the qualitative nature of the research means that findings are exploratory and should be interpreted with caution. Principles have been identified but their efficacy has not been empirically tested. Finally, use of the photo-elicitation method may also entail certain limitations, as the initial selection of images could unintentionally shape or guide participants' interpretations and responses, thereby introducing potential bias into the data collection process.

Future research should aim to corroborate and reaffirm the guiding principles identified here through applied intervention contexts and assess their effectiveness across different cultural and socioeconomic settings. Expanding the sample to include experts from underrepresented regions and conducting longitudinal studies would provide a more comprehensive understanding of the way in which the identified principles influence PA behaviors in other contexts and over time, respectively.

## 5 Conclusions

Present findings underscore the importance of long-term collaboration between people and organisations to promote PA in society through interventions that are based on scientific evidence and well-supported strategies. The purpose of PA promotion interventions must not just be for the target population to enjoy PA and receive the necessary training to be active (motor skill competence, motivation, knowledge of PA and health), but efforts should also be made to create social and physical environments that favour the acquisition of an active lifestyle. All interventions should be embedded within broader social, educational and policy frameworks to strive for greater effectiveness.

In summary, the present report lays the groundwork for future reflections and actions in the field of PA promotion. The findings presented here aim to inspire ongoing dialogue among policymakers, educators, health professionals and researchers, and might contribute to the refinement of strategic principles that can effectively guide the design and implementation of impactful and sustainable interventions. There is strong evidence to support that fostering collective effort will give rise to environments that truly support and facilitate active lifestyles for all.

## Data Availability

The datasets presented in this article are not readily available because uploaded in a hard disk. Requests to access the datasets should be directed to aibar@unizar.es.
